# Two new and one newly recorded species of *Thelcticopis* Karsch, 1884 (Araneae, Sparassidae) from China

**DOI:** 10.3897/zookeys.940.50764

**Published:** 2020-06-11

**Authors:** Yang Zhu, Ye-Jie Lin, Yang Zhong

**Affiliations:** 1 The State Key Laboratory of Biocatalysis and Enzyme Engineering of China, Centre for Behavioural Ecology and Evolution, College of Life Sciences, Hubei University, Wuhan 430062, Hubei, China Hubei University Hubei China; 2 Institute of Zoology, Chinese Academy of Sciences, Beijing 100101, China nstitute of Zoology, Chinese Academy of Sciences Beijing China; 3 School of Nuclear Technology and Chemistry & Biology, Hubei University of Science and Technology, Xianning 437100, Hubei, China Hubei University of Science and Technology Hubei China

**Keywords:** biodiversity, huntsman spiders, Taiwan, taxonomy

## Abstract

Two new species of the genus *Thelcticopis* Karsch, 1884, *T. dahanensis* Zhu & Zhong, **sp. nov.** (♂) and *T. unciformis* Zhu & Zhong, **sp. nov.** (♂), are described and figured from Taiwan Island. *Thelcticopis severa* (L. Koch, 1875) is recorded from Guangdong and Fujian provinces for the first time. So far, *Thelcticopis*, including four species from China, is mainly distributed in the tropical or subtropical areas of China (Hainan, Taiwan, Yunnan, Guangdong, Fujian).

## Introduction

Sparianthinae Simon, 1887 is a subfamily of Sparassidae Bertau, 1872, including 15 genera and 91 described species. The genera included *Decaphora* Franganillo, 1931, *Defectrix* Petrunkevitch, 1925, *Extraordinarius* Rheims, 2019, *Pleorotus* Simon, 1898, *Pseudosparianthis*, *Rhacocnemis* Simon, 1897, *Sagellula* Strand, 1942, *Sampaiosia* Mello-Leitão, 1930, *Sparianthis*, *Stasina*, *Stipax* Simon, 1898, *Strandiellum* Kolosváry, 1934, *Thelcticopis* Karsch, 1884, *Thomasettia* Hirst, 1911, and *Uaiuara* Rheims, 2013 (Rheims and Alayón 2016; Rheims 2019; World Spider Catalog 2020). These are distributed in Latin and South America, Africa, Asia, and Australia. This subfamily can be separated from other sparassid subfamilies by the presence of small retromarginal teeth on the chelicerae, a trilobate membrane with a reduced or inconspicuous median lobe, and male palps bearing a median apophysis (Rheims 2019). Sparianthinae is represented in China by the genera *Sagellula* and *Thelcticopis* (World Spider Catalog 2020).

*Thelcticopis* was proposed by Karsch (1884) as a new name for *Themeropis* L. Kock, 1875, preoccupied by a coleopteran beetle (*Themeropis* Pascoe, 1874). The genus was originally proposed by L. Koch (1875) to include the type species, *T.>T. severa* L. Koch, 1875, described based on a female from China. Years later, Jäger (2005) synonymized *Seramba* Thorell, 1887 with *Thelcticopis* Karsch, 1884, and pointed out that probably the two African species, *Thelcticopis humilithorax* (Simon, 1910) and *T.>T. truculenta* Karsch, 1884, did not belong to *Thelcticopis*. Nevertheless, he kept both species in the genus until a more thorough revision, as discussed in later regional revisions (Jäger 2005; Jäger and Kunz 2005). Currently, *Thelcticopis* includes 48 described species distributed mainly in East, South and Southeast Asia, South and Western Pacific (World Spider Catalog 2020). Two species are reported from China, *T.>T. severa* and *T.>T. zhengi* Liu, Li & Jäger, 2010. While studying new materials collected in Taiwan Island, two new *Thelcticopis* species were recognized and described in the present paper. In addition, we provide new records and photographs of *T.>T. severa*.

## Materials and methods

Specimens were examined and measured with a Leica M205C stereomicroscope. Positions of the tegular appendages are given according to clock positions, based on the left palp in ventral view. Male palps were examined after dissection and detachment from the spiders’ bodies, the epigyna were examined and illustrated after dissection. All photographs were captured with an Olympus C7070 wide zoom digital camera (7.1 megapixels) mounted on an Olympus SZX12 dissecting microscope, and assembled using Helicon Focus 3.10.3 image stacking software. Photographic images were then edited using Adobe Photoshop. Left palps are depicted unless otherwise stated. All specimens are deposited in Centre for Behavioural Ecology and Evolution, College of Life Sciences, Hubei University, Wuhan, China (CBEE).

Leg measurements are shown as: total length (femur, patella, tibia, metatarsus, tarsus). Number of spines is listed for each segment in the following order: prolateral, dorsal, retrolateral, ventral (in femora and patellae ventral spines are absent and fourth digit is omitted in the spination formula). Abbreviations follow Zhong et al. (2017, 2018, 2019):

**ALE** anterior lateral eyes;

**AME** anterior median eyes;

**AW** anterior width of prosoma;

**C** conductor;

**CH** clypeus height;

**dRTA** dorsal branch of RTA;

**E** embolus;

**FD** fertilization duct;

**FE** femur;

**TA** tegular apophysis;

**MS** middle septum;

**Mt** metatarsus;

**OL** opisthosoma length;

**OW** opisthosoma width;

**Pa** patella;

**PL** prosoma length;

**PLE** posterior lateral eyes;

**PME** posterior median eyes;

**Pp** palpus;

**PW** prosoma width;

**RTA** retrolateral tibial apophysis;

**S** spermatheca;

**SP** spermophore;

**Ta** tarsus;

**Ti** tibia. I, II, III, IV–legs I to IV;

**vRTA** ventral branch of RTA.

## Taxonomy

### Family Sparassidae Bertkau, 1872

**Subfamily Sparianthinae Simon, 1897**

#### Genus Thelcticopis

Taxon classificationAnimaliaAraneaeSparassidae

Karsch, 1884

3D8896B0-C1DA-5234-AC52-2F142839F134

##### Type species.

*Thelcticopis severa* (L. Koch, 1875).

##### Diagnosis.

The subfamily Sparianthinae is represented in China by two genera: *Sagellula* Strand, 1942 and *Thelcticopis* Karsch, 1884. However, most species of both genera have been poorly described so far, and the monophyly of these genera is also debatable as *Sagellula xizangensis* (Hu, 2001) may be wrongly placed (Jäger and Yin 2001). Therefore, we just provide a diagnosis between the Chinese *Thelcticopis* and *Sagellula* (only *S. xizangensis*) species in the current paper. The Chinese *Thelcticopis* species are most similar to *S. xizangensis* in having spoon-shaped tegular apophysis in the male palp and median septum in the epigynum (Hu 2001; fig. 187. 1–4), but can be distinguished from the latter by the following characters: 1, tibia of male palp stout, about 1/3 cymbium length in *Thelcticopis*, but normal, less than 1/2 cymbium length in *S. xizangensis*; 2, retrolateral tibial apophysis complicated, branched in most *Thelcticopis* species, but simple in *S. xizangensis*; 3, spermatheca significantly irregular in most *Thelcticopis* species, but globular in *S. xizangensis*; 4, anterior median eye larger than other eyes in *Thelcticopis*, but posterior lateral eyes obviously largest in *S. xizangensis*.

##### Distribution.

Asia and Pacific zoogeographic regions.

#### Thelcticopis dahanensis

Taxon classificationAnimaliaAraneaeSparassidae

Zhu & Zhong sp. nov.

4D014743-FC15-58C9-B145-0957C5541021

http://zoobank.org/B362CCBC-E054-4FA1-B293-07693E07E439

Figures 1 , 5

##### Type materials.

***Holotype*.** ♂ (CBEE), China, Taiwan Island, Pingdong County, Mt. Dahan, 22.41N, 120.74E, 29.VI.2013, J. Liu leg. ***Paratypes*** (CBEE): ♂, same data as holotype.

##### Etymology.

The specific name is a noun in apposition taken from the type locality.

##### Diagnosis.

**Figure 1.**
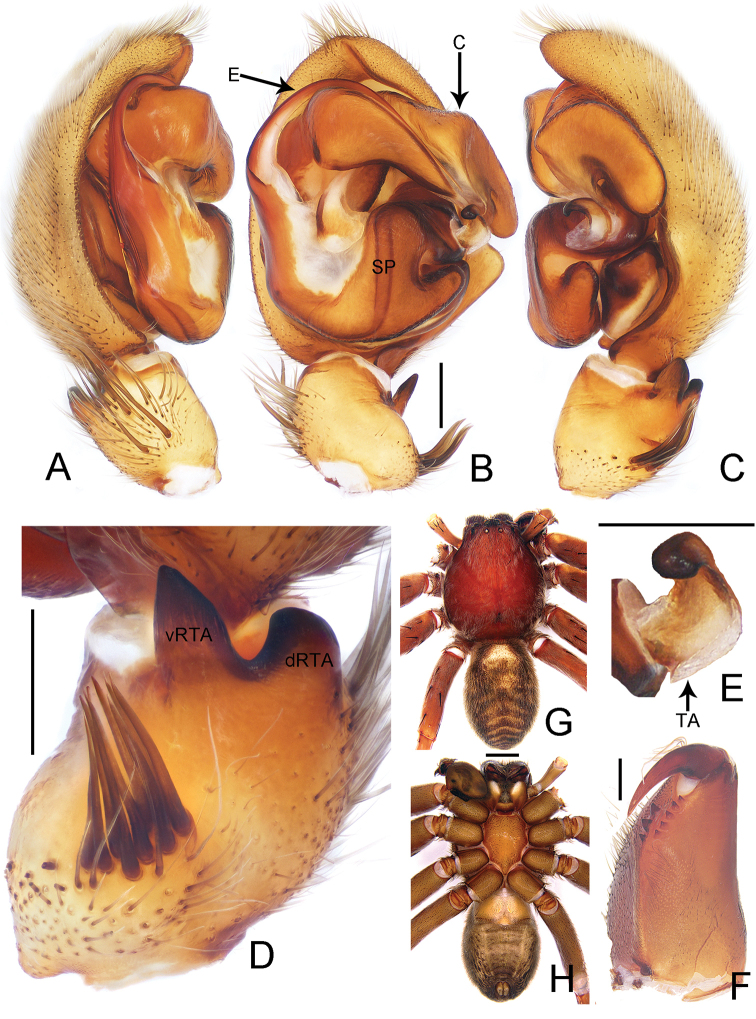
*Thelcticopis dahanensis* Zhu & Zhong, sp. nov., holotype male **A–C** palp, left **D** left male palpal tibia **E** tegular apophysis **F** cheliceral dentition **G, H** male habitus (**A** prolateral view; **B, E, F, H** ventral view; **C, D** retrolateral view; **G** dorsal view). Abbreviations: C–conductor, dRTA–dorsal branch of RTA, E–embolus, TA–tegular apophysis, SP–spermophore, vRTA–ventral branch of RTA. Scale bars: 0.5 mm (**A–F**); 5 mm (**G, H**).

The male of this new species resembles those of other Chinese *Thelcticopis* species (*T.>T. severa*, *T. unciformis* sp. nov. and *T.>T. zhengi*) in having stout tibia, broad cymbium and spoon-shaped tegular apophysis, but can be separated from *T.>T. severa* by RTA arising distally from tibia, branched (arising proximally, not branched in *T.>T. severa*); from *T. unciformis* sp. nov. by the developed conductor with two branches distally, dorsal branch extending beyond ventral one (dorsal branch not extending beyond ventral one in *T. unciformis* sp. nov.), from *T.>T. zhengi* by the long embolus with filiform end, visible in ventral view (but short, with blunt end, covered by a large tegular apophysis in *T.>T. zhengi*) (Fig. 1A–E).

##### Description.

**Male.**PL 6.1, PW 5.3, AW 2.8, OL 6.0, OW 3.1. Eyes: AME 0.25, ALE 0.21, PME 0.18, PLE 0.20, AME–AME 0.25, AME–ALE 0.34, PME–PME 0.58, PME–PLE 0.69, AME–PME 0.36, ALE–PLE 0.34, CHAME 0.16, CHALE 0.14. Spination: Palp: 131, 101, 0002; Fe: I–II 323, III 322, IV 321; Pa: I–IV 000; Ti: I–II 212 10, III–IV 2126; Mt: I–II 1012, III 3032, IV 3034. Measurements of palp and legs: Palp 7.5 (2.2, 0.8, 1.3, –, 3.2), I 20.6 (5.6, 2.8, 5.6, 5.3, 1.3), II 18.7 (5.1, 2.7, 5.0, 4.7, 1.2), III 15.5 (4.6, 2.3, 3.9, 3.7, 1.0), IV 19.7 (5.8, 2.2, 4.8, 5.7, 1.2). Leg formula: I-IV-II-III. Cheliceral furrow with three anterior and five posterior teeth, without denticles (Fig. 1F). Dorsal prosoma reddish brown, posterior margins dark, with shallow fovea and radial furrows. Chelicerae deep reddish brown. Sternum yellowish brown, with margin deep brown. Gnathocoxae and labium deep yellowish brown, with white distal lips. Legs deep reddish to yellowish brown, covered by short spines. Dorsal opisthosoma with irregular patches and distinct median chevrons in posterior half. Ventral opisthosoma with patches especially in lateral half (Fig. 1G, H).

Palp as in diagnosis. Cymbium approximately two times longer than tibia in ventral view. Conductor arising from tegulum in the 11-o’clock-position. Appendage of median apophysis finger-shaped in ventral view. Sperm duct almost straight in ventral view. vRTA with tip pointed and dRTA blunt in retrolateral view. Palpal tibia retrolaterally with distinct bunch of nine setae (Fig. 1A–E).

**Female.** Unknown.

##### Distribution.

Known only from the type locality (Fig. 5).

#### Thelcticopis severa

Taxon classificationAnimaliaAraneaeSparassidae

(L. Koch, 1875)

C37B32BB-E1F7-5640-BC72-9B86C263B7B0

Figures 2 , 3 , 5

Themeropis severa L. Koch, 1875: 699, pl. 60, fig. 1 (♀).
Thelcticopis severa Simon, 1897: 72 (transferred from Themeropis).

##### Remarks.

See the World Spider Catalogue for the full list of references.

##### Material examined.

2♂, 1♀ (CBEE), China, Hainan Island, Wuzhishan National Reserve, 18.89N, 109.69E, 29.VI.2013, F.X. Liu leg.; 2♂, 1♀ (CBEE), China, Shenzhen City, Bijiashan Park, 22.56N, 114.08E, 26.VII.2018, Q.L. Lu leg; 2♂ (CBEE), China, Fujian Province, Wuyishan National Reserve, 27.58N, 117.48E, 25.VIII.2019, Y. Zhong leg.

##### Diagnosis.

**Figure 2.**
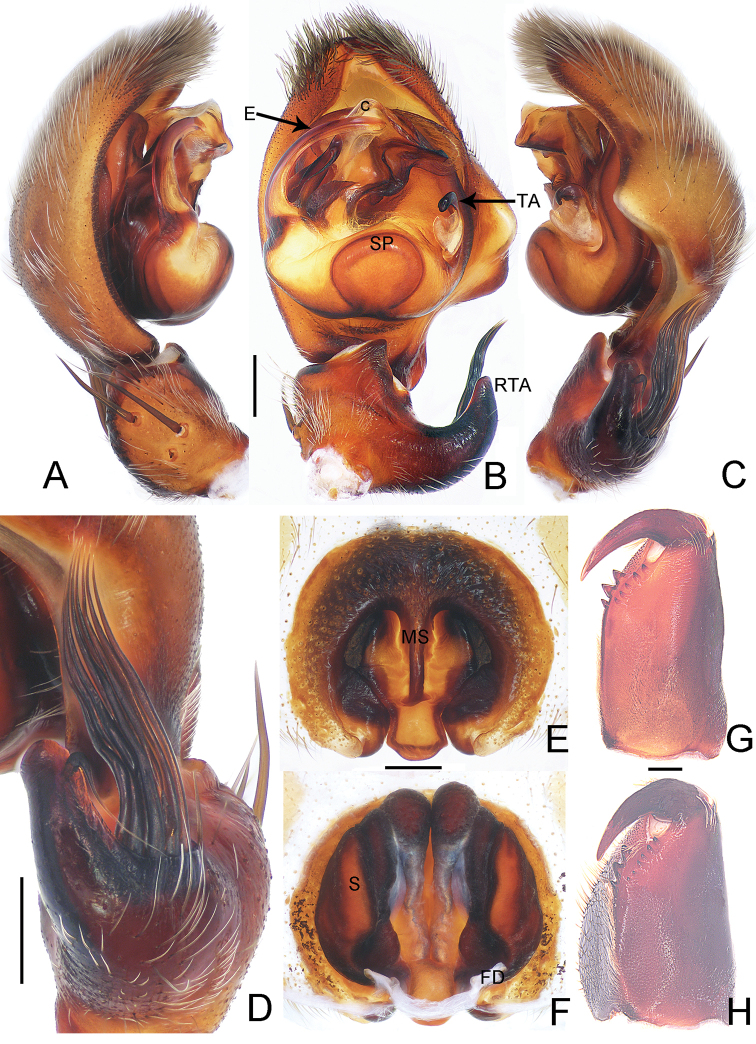
*Thelcticopis severa* (L. Koch, 1875) **A–C** palp, left **D** left male palpal tibia **E** epigyne **F** vulva **G, H** cheliceral dentition (**A** prolateral view; **B, E, G, H** ventral view; **C, D** retrolateral view; **F** dorsal view; **G** male; **H** female). Abbreviations: C–conductor, E–embolus, FD–fertilization duct, MS–middle septum, RTA–retrolateral tibial apophysis, S–Spermatheca, SP–spermophore, TA–tegular apophysis. Scale bars: 0.5 mm

Males of this species can be distinguished from males of other *Thelcticopis* species by its unique bases of RTA with seven or eight stiff setae, long and standing in line (almost the same length as dRTA) and tip of RTA with one stiff seta bending backwards in retrolateral view (Fig. 2A–D). Females are similar to those of *Thelcticopis picta* (Thorell, 1887) in having median septum somewhat heart-shaped with a tongue-like posterior structure pointing in the direction of the epigastric furrow (Jäger 2005: figs 1–7), but distinguished from the latter by the following characters: anterior part of the median septum with a longitudinal ridge (absent in *T. picta*); ends of internal duct system inconspicuous in dorsal view (visible in *T. picta*) (Fig. 2A–F).

##### Description.

See Hu and Ru (1988) and Yin et al. (2012).

**Figure 3.**
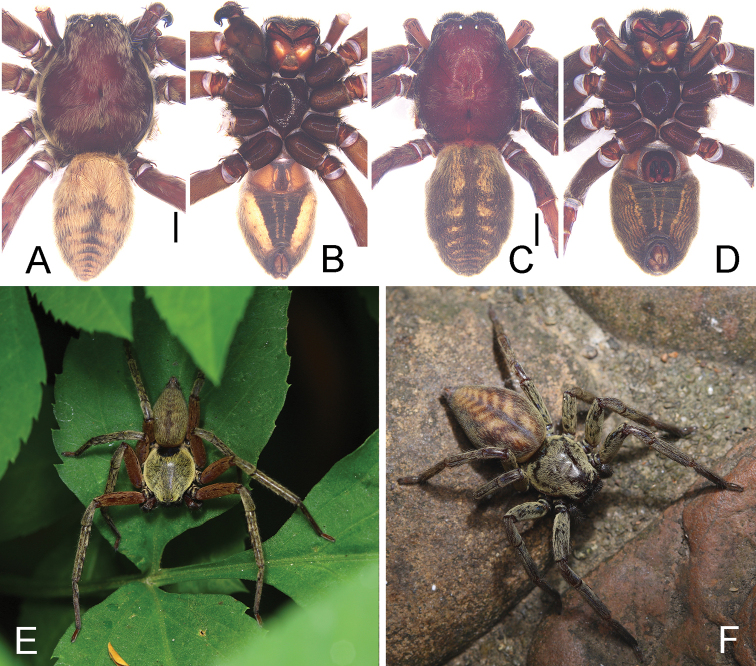
**A–D** habitus of *Thelcticopis severa* (L. Koch, 1875) **A** male, dorsal **B** male, ventral **C** female, dorsal **D** female, ventral **E, F** photographs of living *Thelcticopis severa* (L. Koch, 1875) from Bijiashan park **E** male **F** female. Photographs by Qianle Lu. Scale bars: 5 mm.

##### Distribution.

China (Guangdong, new province record; Guangxi; Hainan; Fujian, new province record; Hongkong; Hunan; Taiwan; Yunnan; Zhejiang); Korea; Japan; Laos (Fig. 5).

#### Thelcticopis unciformis

Taxon classificationAnimaliaAraneaeSparassidae

Zhu & Zhong sp. nov.

18214BC4-154B-52B8-AB6B-BC54C3D2EE95

http://zoobank.org/A2035161-2D57-493D-8E14-5E12A5357C52

Figures 4 , 5

##### Type materials.

***Holotype*.** ♂ (CBEE), China, Taiwan Island, Taipei City, Mt. Yangming, 25.17N, 121.53E, 5.VII.2013, J. Liu leg. ***Paratypes*** (CBEE): ♂, same data as holotype.

##### Etymology.

The specific name is derived from Latin adjective *unciformis*, -*is*, -*e*, meaning hooked and referring to the embolus being curved.

##### Diagnosis.

**Figure 4.**
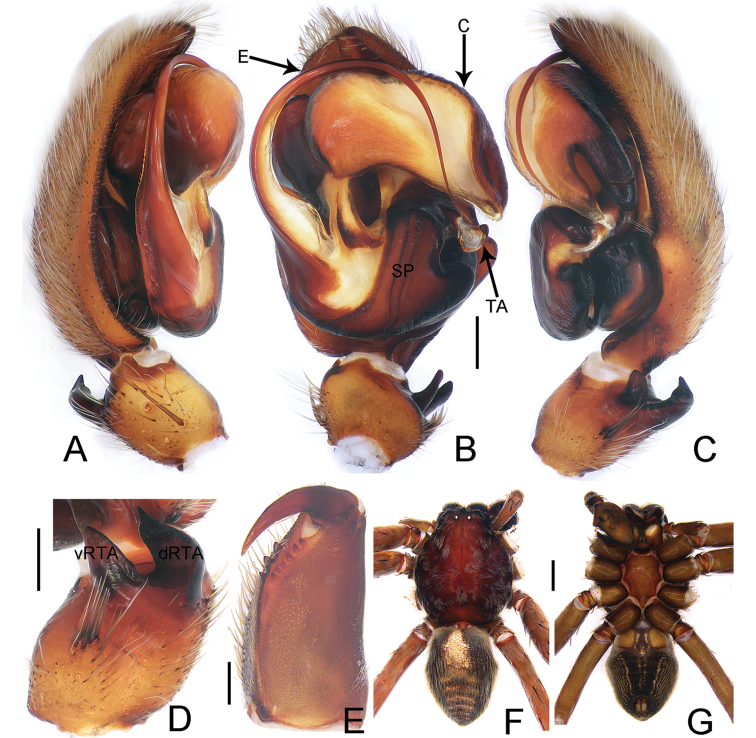
*Thelcticopis unciformis* Zhu & Zhong, sp. nov., holotype male **A–C** palp, left **D** left male palpal tibia **E** cheliceral dentition **F, G** male habitus (**A** prolateral view; **B, E, G** ventral view; **C, D** retrolateral view; **F** dorsal view). Abbreviations: C–conductor, dRTA–dorsal branch of RTA, E–embolus, TA–tegular apophysis, SP–spermophor, vRTA–ventral branch of RTA. Scale bars: 0.5 mm (**A–E**); 5 mm (**F, G**).

The male of *T. unciformis* resembles that of *T. dahanensis* (Fig. 1A–D) by the embolus arising from the tegulum at the 8:30 to 9-o’clock position, embolus tip slender; tibia with retrolateral setae, RTA arising distally from the tibia. However, it can be distinguished by the embolus tip extending beyond the conductor (not so in *T. dahanensis*); dRTA tip pointed in retrolateral view (blunt in *T. dahanensis*) (Fig. 4A–D).

##### Description.

**Figure 5.**
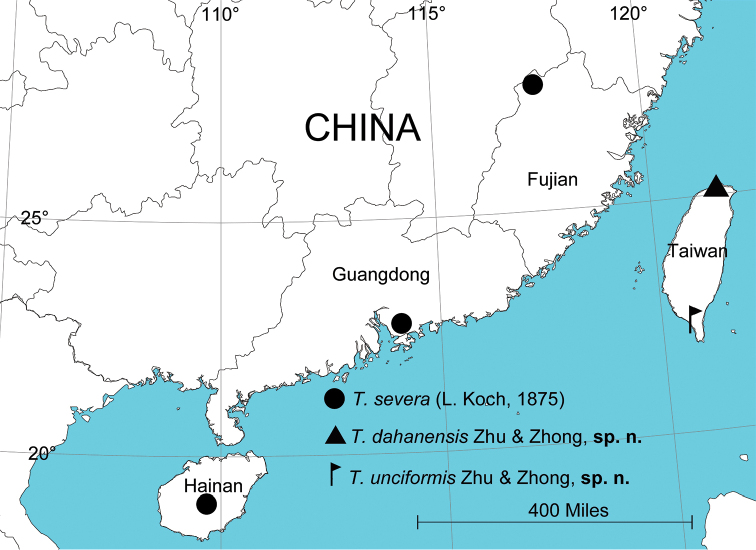
Collection localities of three *Thelcticopis* species from China.

**Male.**PL 7.5, PW 6.0, AW 3.3, OL 7.1, OW 4.0. Eyes: AME 0.39, ALE 0.30, PME 0.21, PLE 0.27, AME–AME 0.21, AME–ALE 0.35, PME–PME 0.58, PME–PLE 0.77, AME–PME 0.33, ALE–PLE 0.32, CHAME 0.15, CHALE 0.12. Spination: Palp: 131, 101, 0002; Fe: I–III 323, IV 321; Pa: I–IV 000; Ti: I–II 212(10), III 2026, IV 2226; Mt: I–II 1012, III 1014, IV 3034. Measurements of palp and legs: Palp 7.3 (2.0, 0.8, 1.2, –, 3.3), I 22.9 (6.2, 3.1, 6.3, 5.8, 1.5), II 21.3 (6.1, 2.9, 5.7, 5.2, 1.4), III 17.6 (5.4, 2.6, 4.2, 4.1, 1.3), IV 22.4 (6.8, 2.4, 5.6, 6.1, 1.5). Leg formula: I-IV-II-III. Cheliceral furrow with three anterior and six posterior teeth, without denticles (Fig. 5C). Dorsal prosoma deep reddish brown, posterior margins dark, with shallow fovea and radial furrows. Chelicerae deep reddish brown. Sternum yellowish to reddish brown, with margin reddish brown. Gnathocoxae deep yellowish brown, with white distal lips. Labium deep reddish brown. Legs deep yellowish brown, covered by short spines. Dorsal opisthosoma with irregular patches and distinct median chevrons in posterior half. Ventral opisthosoma yellowish brown, with larger and black hairs (Fig. 4F, G).

Palp as in diagnosis. Cymbium about three times longer than tibia in ventral view. Conductor arising from tegulum in an 11-o’clock-position. Median apophysis spoon-shaped and bifid in ventral view. Sperm duct slightly curved in ventral view. vRTA and dRTA with pointed tips in retrolateral view. Palpal tibia retrolaterally directed with distinct bunch of six setae (Fig. 4A–D).

**Female.** Unknown.

##### Distribution.

Known only from the type locality (Fig. 5).

## Supplementary Material

XML Treatment for Thelcticopis

XML Treatment for Thelcticopis dahanensis

XML Treatment for Thelcticopis severa

XML Treatment for Thelcticopis unciformis
